# Effects of prenatal exposure to NO_2_ on children’s neurodevelopment: a systematic review and meta-analysis

**DOI:** 10.1007/s11356-020-08832-y

**Published:** 2020-04-30

**Authors:** Li Shang, Liren Yang, Wenfang Yang, Liyan Huang, Cuifang Qi, Zixuan Yang, Zhuxuan Fu, Mei Chun Chung

**Affiliations:** 1 Department of Obstetrics and Gynecology, Maternal & Child Health Center, The First Affiliated Hospital of Xi’an Jiaotong University, No. 277, Yanta West Road., Xi’an, 710061 Shaanxi Province People’s Republic of China; 2 School of Public Health, Xi’an Jiaotong University Health Science Center, Xi’an, Shaanxi People’s Republic of China; 3 Antai College, Shanghai Jiao Tong University, Shanghai, People’s Republic of China; 4 Graduate School of Public Health, University of Pittsburgh, Pittsburgh, PA USA; 5 Department of Public Health and Community Medicine, Tufts University School of Medicine, Boston, MA USA

**Keywords:** Prenatal exposure, Nitrogen dioxide, Psychomotor, Cognition, Neurodevelopment, Children

## Abstract

The neurotoxicity of NO_2_ exposure is well-known and potentially causes impaired of neural functions. This review aimed to estimate associations between prenatal NO_2_ exposure and neurodevelopment for children. Articles published until May 2019 reported prenatal NO_2_ exposure and children’s cognition, psychomotor, language, attention, IQ, and behavior function were searched according to all related terms. The main databases we retrieved included PubMed, Web of Science, Embase, and Cochrane Library. Coefficient was extracted, conversed, and synthesized by random effects meta-analysis. Meanwhile, qualitatively describe would be used for some studies which cannot be synthesized quantitatively for lack of quantity or methods inconsistency. Finally, a total of 3848 citations were searched, and only 10 studies were included. We estimated that per 10 μg/m^3^ increase of NO_2_ during pregnancy was associated with a − 0.76 point decrease in global psychomotor (95% CI, − 1.34, − 0.18) and a − 0.62 point decrease in fine psychomotor for children (95% CI, − 1.09, − 0.16). But no significant association found in general cognitive and language. In addition, through the literature review, it seemed that prenatal exposure to NO_2_ might cause adverse impacts on children’s attention, IQ, and different behaviors, but this requires confirmation from further researches. Our study indicated that prenatal exposure to NO_2_ seems to be associated with impaired neural development for children, especially for fine psychomotor. However, further studies are needed for determining the effects of prenatal air pollution exposure on attention, IQ, and behavior.

**Electronic supplementary material:**

The online version of this article (10.1007/s11356-020-08832-y) contains supplementary material, which is available to authorized users.

## Introduction

Ambient air pollution is recognized as a global issue due to its adverse impacts on air quality and public health. According to statistics, the exposure concentration of air pollutants in many developed countries and most of developing countries were considerably higher than the guidelines recommended by the World Health Organization (WHO) (Organization 2007). Among them, nitrogen dioxide (NO_2_), a traffic-related air pollutant mainly derived from automobile exhaust and fuel combustion, still shows excessive exposure levels in many countries and even continues to increase. According to statistics, the exposure level of NO_2_ had increased 2.7 times from 1996 to 2012 in China (Seltenrich 2016).

The embryo and fetus, as the most sensitive period, are extremely susceptible to ambient NO_2_ exposure. Maternal exposure to NO_2_ can induce some abnormal reactions, including inflammation reaction, oxidative stress, and DNA methylation, which may cause adverse effects on organs and tissue of the offspring (Fiorito et al. 2018; Mirowsky et al. 2016). Some studies reported that maternal exposure to oxynitride might cause the oxidative damage of brain white and gray matter through oxidative stress (Anderson et al. 2018; Nagiah et al. 2015; Akhtar et al. 2017; Murray et al. 2015; Tonni et al. 2014). In addition, it also suggested that exposure to NO_2_ caused disruption of BBB and accumulation of amyloid β42 and α-synuclein starting in childhood through neural inflammation (Allen et al. 2014; Calderon-Garciduenas et al. 2015). Based on those evidences, it can be inferred that maternal exposure to NO_2_ may have adverse effects on children’s neural development.

Some studies and reviews have supported the hypothesis that exposure to ambient NO_2_ and other air pollution was associated with an increased risk of autism spectrum disorder (ASD) (Chun et al. 2020; Flores-Pajot et al. 2016) and attention deficit hyperactivity disorder (ADHD) for children (Donzelli and Carducci 2019; Donzelli et al. 2019; Forns et al. 2018; Min and Min 2017). However, the effects of prenatal exposure to NO_2_ on children’s neurodevelopment (including cognitive, psychomotor, language, and behavioral functions) are still unclear for different conclusions in related studies (Ren et al. 2019; Porta et al. 2016; Sentis et al. 2017). A study based on six European birth cohorts found that NO_2_ exposure during pregnancy was associated with delayed fine psychomotor development during childhood, but not cognition (Guxens et al. 2014). Nevertheless, some related studies did not find significant association between maternal exposure to NO_2_ and psychomotor development (Lin et al. 2014), and some studies considered it to have adverse effects on children’s cognitive function (Guxens et al. 2014; Porta et al. 2016). And for behavior, different studies also have drawn different conclusions (Ren et al. 2019; Yorifuji et al. 2017).

However, to our knowledge, there are no systematic reviews specifically addressing the association of prenatal NO_2_ exposure and children’s neural development. Therefore, we systematically review related literatures to explore and assess the incremental effect of 10 μg/m^3^ exposure to NO_2_ during gestation on children’s neurodevelopment, including cognition, language, fine and gross psychomotor, behavior, IQ, attention, and emotions.

## Materials and methods

This review was developed in accordance with the PRISMA statement for systematic reviews (www.prisma-statement.org) and was registered with PROSPERO (www.crd.york.ac.uk/PROSPERO) under protocol number CRD42019125057.

### PECO question

The research question was determined using the PECO strategy: population (children having undergone neurodevelopment evaluations and their mothers); exposure (10 μg/m^3^ exposure to NO_2_ during gestation); comparison (10 μg/m^3^ incremental increase); and outcome (children’s neurodevelopment, including cognition, fine and gross psychomotor, behavior, IQ, language, and attention). Based on this, we established research question as follows: For children, what is the incremental effect of 10 μg/m^3^ exposure to NO_2_ during gestation on children’s neurodevelopment, including cognition, language, fine and gross psychomotor, behavior, IQ, attention, and emotions.

### Search strategy

To identify studies that estimated the effects of maternal exposure to NO_2_ on children’s neurodevelopment, related articles published until May 12, 2019, were searched using PubMed, Web of Science, Embase, and Cochrane Library. All related terms were used for retrieval, such as “maternal,” “air pollution,” “traffic-related pollution,” “nitrogen dioxide,” “neurodevelopment,” “cognition,” “psychomotor,” “language,” “behavior,” “attention,” and “child.” Full details were provided in the Appendix A. In addition, we manually searched the references in each included studies for additional publications.

### Selection criteria and data collection

Studies with quantitative data on associations between maternal exposure to NO_2_ and children’s neurodevelopment were considered. Research articles were included if they (1) were written in English;( 2) were cohort or cross-sectional studies in human subjects; (3) measured maternal exposure to ambient NO_2_ throughout pregnancy or in trimester-specific periods; and (4) assessed children’s neurodevelopment outcomes such as cognition, language, psychomotor, emotions, behavior, emotions, and IQ levels. In addition, since our review only focused on the effects of maternal exposure to ambient NO_2_ on children’s development of neural function, some studies were excluded if (1) exposure window outside of pregnancy period and (2) outcome variables only included neurodevelopmental disorders, such as autistic spectrum disorder and attention deficit hyperactivity disorder.

The abstracts and titles screening, full-text screening, and data extraction were all carried out by two investigators independently. Discrepancies between the two investigators were resolved by discussion. The data extracted from each paper included study design, location, study period, sample size, methods and measurement, covariates, statistical analysis, and the ORs and 95% CIs used during statistical analyses.

### Risk-of-bias assessment

The risk-of-bias (ROB) for each included study was assessed using a modified instrument specifically for examining associations between exposure to multiple air pollutants and autism spectrum disorder (Lam et al. 2016). This tool was developed based on the Cochrane Collaboration’s risk-of-bias tool (McHenry et al. 2018) and assesses the following 9 ROB domains: source population representation, blinding, exposure assessment (for air pollutants), outcome assessment, potential cofounding, incomplete outcome data, selective outcome reporting, conflict of interest, and other biases. Since the instructions for the outcome assessment domain were not fit for our review, prior to assessing ROB, we had modified the evaluation standard and instructions of outcome variable according to the criteria for the quality of the neuropsychological assessments created by McHenry et al. We rated each ROB domain as “low,” “probably low,” “probably high,” or “high” risk of bias or “not applicable” (risk of bias area not applicable to study) according to specific criteria as described in the modified ROB instruments (Appendix B). All included papers were independently evaluated by two investigators, and the contradictions were resolved through discussion.

### Data synthesis and analysis

Meta-analysis was a preferred synthetic method when two or more unique studies reporting the same outcomes and provided sufficient quantitative data for meta-analysis. Prior to conducting the meta-analysis, coefficients and their confidence intervals (CIs) for the associations between NO_2_ exposure and outcome variables were extracted for meta-analysis. And standard errors (SEs) of the coefficients were calculated from the reported CIs under normal distribution assumption. And then the coefficients and SEs were converted to the same unit of 10 μg/m^3^ increase in NO_2_, which facilitate the consolidation of estimate from different studies. Increase of part per billion (ppb) was conversed to μg/m^3^ under standard atmosphere (101.325 Kpa) and standard temperature (25 °C), and the specific process of data conversion was shown in Appendix C. D-L random effects model meta-analysis was performed to merge effect value. And heterogeneity was quantified by I^2^ statistics. Meta-analysis was performed using “metafor” package in R version 3.5.2. Significance level was set at *p* < 0.05.

While other outcome variables were too heterogonous to meta-analysis, synthesized qualitatively in narrative was performed in accordance with the document Guidance on the Conduct of Narrative Synthesis in Systematic Reviews (Rodgers et al. 2019). In this process, we used tabulation and visual representations of data to simplify the key characteristics of included studies. And then, we synthesized evidence narratively. In the end, we compared our narrative results to those of other systematic reviews to judge the robustness of the results of this study.

## Results

### Search results

**Fig. 1:**
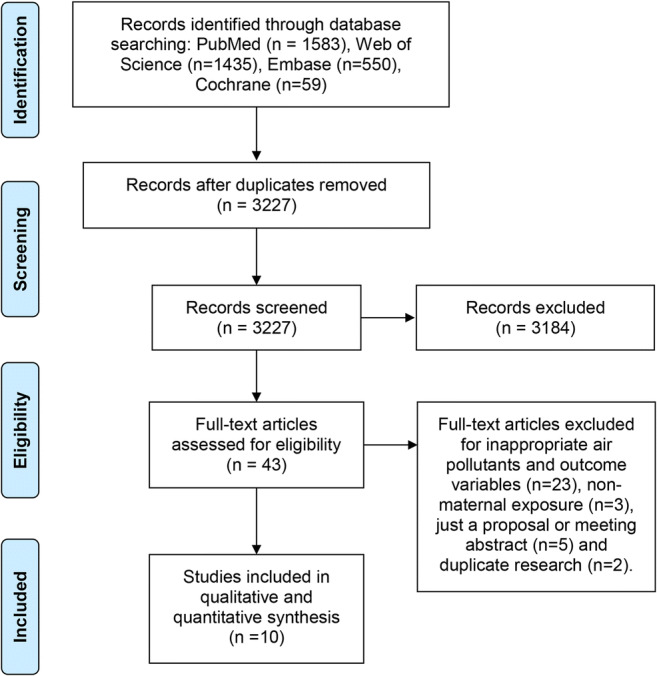
Flowchart of search strategy and selection of studies for inclusion in review

A total of 3848 citations (excluding duplicates, *n* = 647) were retrieved in our review, and 43 records were screened for full-text based on our study eligibility criteria independently. Finally, a total of 10 citations were included in this systematic review. The flowchart of the study selection process is depicted in Fig. 1.

### Characteristics of included studies

All of 10 studies were published between 2012 and 2019. Table 1 presents the characteristics of included studies. Ages of children ranged from 6 months to 8 years of age. Sample sizes ranged from 438 (Lertxundi et al. 2015) to 33,890 (Yorifuji et al. 2017). In addition to one cross-sectional study (Ren et al. 2019) and one longitudinal survey (Yorifuji et al. 2017), the remaining 8 studies were based on prospective birth cohort design. And 5 studies were conducted in Europe (Guxens et al. 2012, 2014; Lertxundi et al. 2015, 2019; Sentis et al. 2017), such as Italy and Spanish, while the remaining 5 studies were conducted separately in Asian countries including China (Lin et al. 2014; Porta et al. 2016; Ren et al. 2019), South Korea (Kim et al. 2014), and Japan (Yorifuji et al. 2017). In particular, Guxens et al. (Guxens et al. 2014) had published a study which analyzed data from 6 European population-based birth cohorts conducted in 11 different regions, and the effect values for each subproject were provided in the supplemental material. So those 11 subprojects were also included in our meta-analysis.

**Table 1:** Characteristics of included studies

**First author, year**	**Location**	**Study design**	**Study period**	**Age of child**	**No.**^**a**^	**Exposure assessment**	**Outcome variables**	**Outcome measurement**	**Method of outcome measurement**	**Exposure distribution**	**Main conclusion**
Guxens, 2014	Six European countries (the Netherlands, Germany, France, Italy, Greece, Spain)	Birth cohorts	1997–2008	0–6 years of age	9482	LUR models	General cognition; language; global psychomotor; fine psychomotor; gross psychomotor	Different scales	Neuropsychological tests administered by psychologists or pediatricians or by questionnaires answered by the parents	Median, 11.5–43.9 μg/m^3^	Global psychomotor development score was reduced by 0.68 points (95% CI = − 1.25 to − 0.11), and fine psychomotor development score was reduced by 0.67 points (95% CI = − 1.31 to − 0.03) for each 10 μg/m^3^ increase in NO_2_. But no significant association was found in NO_2_ exposure and general cognition, language, and gross psychomotor
Lertxundi, 2015	Spanish-Guipúzcoa	Perspective birth cohorts	From May 2006 to January 2008	13–18 months of age	438	LUR models	Motor score; mental score	Bayley Scales of Infant Development (BSID)	All testing was carried out in healthcare centers by one of two specially trained neuropsychologists who were blinded to the child’s exposure status. They also applied a strict protocol, including training sessions in which inter-observer differences were discussed	Mean ± SD, 20.3 ± 6.6 μg/m^3^; range, 8.06–44.6 μg/m^3^	A 1 μg/m^3^ increase in NO_2_ was associated with a significant decrease of − 0.29 points in mental scale (− 0.47, − 0.11; *p* = 0.008) and a decrease of − 0.14 point in motor scale (− 0.34; 0.06; *p* = 0.259)
Yorifuji, 2017	Japan	Longitudinal survey	2001–2015	8 years of age	33,890	The exposure level of pregnant women was represented by municipality-representative monthly average concentrations	Interrupting others; Destroying toys and/or books; hurting other people; inability to wait his/her turn during play; causing public disturbance; failure to pay attention when crossing a street; lying	Child Behavior Checklist/4–18 Japanese Edition	All questionnaires were mailed to participant to fill in and return	Born in January 2001: mean ± SD, 15.7 ± 7.4 ppb; range, 4.6–1231.1 ppb; Born in July 2001: mean ± SD, 17.7 ± 8.0 ppb; range, 4.6–1231.1 ppb	A one-IQR increase (10.8 ppb) in NO_2_ exposure was associated with failure to pay attention when crossing a street (adjusted OR = 1.10; 95% CI, 1.02–1.19), but no significant associations were found in other behavior development
Ren, 2019	China-Wuhan	Cross-sectional study	October to December 2017	3–4 years of age	397	Utilizing the air pollution concentrations in kindergartens as the surrogate of maternal exposure during pregnancy	Behavioral problems: total difficulties; emotion symptoms; conduct symptoms; hyperactivity/inattention; peer relationship problems; pro-social behavior	Strengths and Difficulties Questionnaire (SDQ)	Teachers in three kindergartens sent questionnaires to children and asked their parents to fill out and return them to kindergartens within 1 week	Mean ± SD, 49.9 ± 5.5 μg/m^3^; range, 37.1–59.3 μg/m^3^	In single-pollutant models, positive association was observed between exposure to NO_2_ and total difficulties (aOR = 1.204; 95 % CI, 1.042, 1.392), especially in the first trimester with aOR = 1.039 (95% CI, 1.013, 1.066). But no significant associations were found in NO_2_ exposure and other behavior problems
Sentís, 2017	Seven regions of Spanish	Prospective birth cohorts	2003–2008	4–5 years of age	1298	LUR models	Attentional function: hit reaction time(HRT); the standard error of the hit reaction time (HRT(SE)); the number of omission errors; the number of commission errors; the detectability or attentiveness (d’)	The 2nd edition of Conners Kiddie Continuous Performance Test (K-CPT)	Children were individually tested with computer in a quiet room by trained investigator.	Mean ± SD, 31.1 μg/m^3^; range, 19.5–35.2 μg/m^3^	It found that per 10 μg/m^3^ increase in prenatal NO_2_ was associated with HRT (SE) (Coef = 1.12; 95% CI, 0.22–2.02) and increased omission errors (Coef = 1.06; 95% CI, 1.01–1.11). And the associations between pre- and postnatal NO_2_ exposure and omission errors were predominantly observed in girls
Porta, 2016	Italian-Rome	Prospective birth cohorts	2003–2010	7 years of age	465	LUR models	Verbal language: verbal IQ; performance IQ; full-scale IQ. Performance score: verbal comprehension index (VCI); perceptual organization index (POI); freedom from distractibility index (FDI); processing speed index (PSI)	Wechsler Intelligence Scale for Children-III edition (WISC-III)	It was administered at the child’s home by three specially trained psychologists, who were unaware of the aim of the study and children’s exposure levels	Mean ± SD, 44.9 ± 10 μg/m^3^; range, 22.5–85.1 μg/m^3^	A 10 μg/m^3^ higher NO_2_ exposure during pregnancy was associated with 1.4 fewer points (95% confidence interval = − 2.6, − 0.20) of verbal IQ, and 1.4 fewer points (95% confidence interval = − 2.7, − 0.20) of verbal comprehension IQ
Lin, 2014	China -Taiwan	Prospective cohort study	From October 2003 to January 2004	6 months and 18 months of age	533	Being linked from the air-quality monitoring stations of town	Gross motor; fine motor; language; social/self-care abilities	Taiwan Birth Cohort Pilot Study (TBCS) scale	The TBCS scale is a parent-reported measure of a child’s neurodevelopmental performance and can be easily completed by the majority of parents	Mean ± SD, 18.2 ± 5.6 μg/m^3^; range, 6.4–28.3 μg/m^3^	In this study, NO_2_ exposure was not significantly associated with children’s gross motor, fine motor, language, and total neurobehavioral developmental scores
Kim, 2014	South Korea	Prospective cohort study	From 1st of January 2006 to 31st of December 2008	6 months, 12 months, and 24 months of age	520	Inverse distance weighting (IDW)	Mental developmental index (MDI); psychomotor developmental index (PDI)	Bayley Scale of Infant Development II (K-BSID-II)	Trained examiners at each center conducted the test for 30 to 45 min in a quiet room. Training of examiners was coordinated by a specialist	Mean ± SD, 26.3 ± 8.4 μg/m^3^; range, 13.1–15.1 μg/m^3^	Maternal NO_2_ exposure was related with impairment of psychomotor development (β = − 1.30; *p* = 0.05) but not with cognitive function (β = − 0.84; *p* = 0.20). In a multiple linear regression model, there were significant effects of prenatal NO_2_ exposure on MDI (β = − 3.12; *p* < 0.001) and PDI (β = − 3.01; *p* < 0.001) at 6 months, but no significant association was found at 12 and 24 months of age
Guxens, 2012	Four regions of Spanish	Prospective cohort study	2003–2008	14 months of age (rang: 11–23 months)	1889	LUR models	Mental development	Bayley Scales of Infant Development	All testing was done in the healthcare center in the presence of the mother, by 12 specially trained psychologists	Mean ± SD, 29.0 ± 11.2 μg/m^3^	It was found no associations between NO_2_ exposure and mental development [β (95% CI) = − 0.95 (− 3.90, 1.89). But strong inverse associations were estimated for NO_2_ and mental development among infants whose mothers reported low intakes of fruits/vegetables during pregnancy [− 4.13 (− 7.06, − 1.21) for a doubling of NO_2_]
Lertxundi, 2019	Three regions of Spanish	Prospective cohort study	From February 2004 to February 2008	4–6 years	1119	LUR models	Verbal; perceptive-manipulative; numeric; memory; Motor (gross and fine); general cognitive index (GCI)	McCarthy Scales of Children’s Abilities (MSCA)	All testing was performed in health centers by the neuropsychologists with training	Mean ± SD, 32.3 ± 0.4 μg/m^3^	These findings suggest a sex-dependent effects on neuropsychological development at 4–6 years of age, with a greater vulnerability in boys, specifically in domains related to memory, verbal, numeric ,and general cognition

LUR, land use regression; SD, standard difference; IQR, interquartile range

^a^Number of subjects with NO_2_ exposure and neural development available, which were included in final analysis

### Risk of bias assessment

**Fig. 2:**
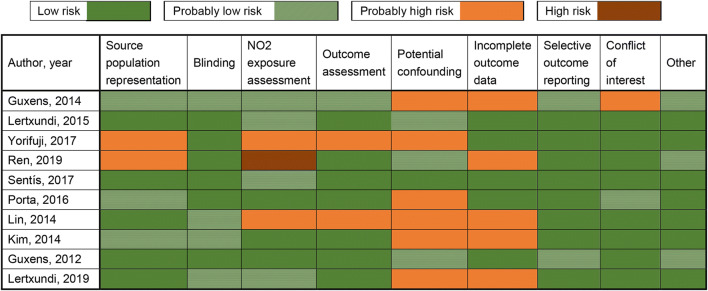
Risk of bias of each study and summary chart for including articles

Overall, most studies were rated as “low” or “probably low” ROB in most domains other than “potential confounding” and “selective outcome reporting”(Fig. 2**)**. The incomplete outcome data in half of the studies were not well described and not adequately addressed. Otherwise, 6 studies were considered as “probably high” ROB for potential confounding that failed to adjust for many of the important confounders, such as maternal IQ which is significantly associated with children’s neurodevelopment (Kim et al. 2014). It is worth noting that one study was rated as “high” ROB in NO_2_ exposure assessment, because it utilized the air pollution concentrations in kindergarten as the surrogate of maternal exposure during pregnancy.

### Meta-analysis

After preliminary synthesis, we found that studies involving general cognition, language, global psychomotor, fine psychomotor, and gross psychomotor could be quantitatively synthesized.

#### General cognitive

**Fig. 3:**
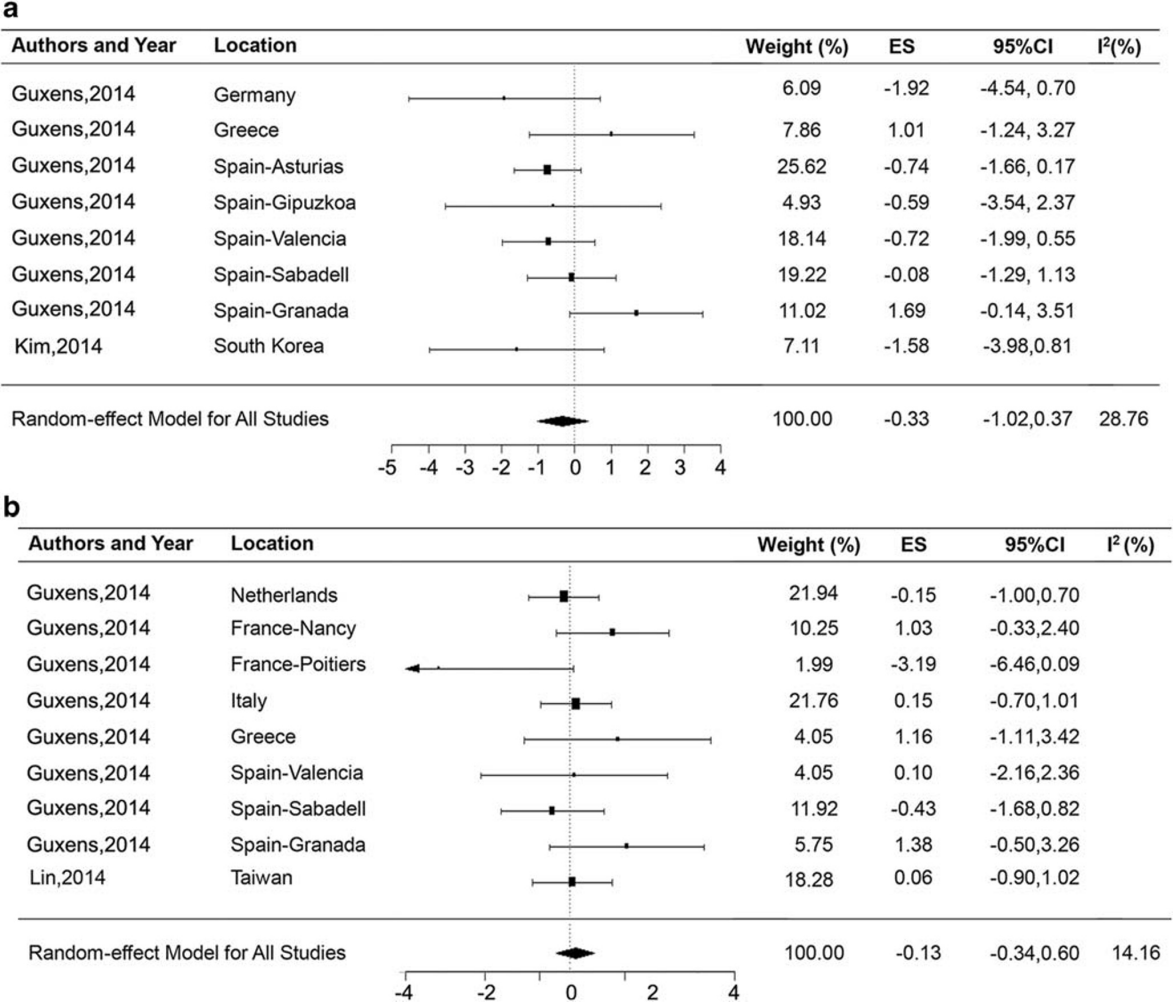
Meta-analysis of maternal exposure to NO_2_ on children’s general cognition (**a**) and language (**b**). ES, effect size; CI, confidence interval; *I*^2^ = percentage of the total variability due to between-areas heterogeneity

Two citations (Guxens et al. 2014; Kim et al. 2014) (including 8 cohort studies from different regions) estimated the effect of maternal exposure to NO_2_ on children’s general cognition. Overall, it showed that a 10 μg/m^3^ increase in maternal NO_2_ exposure was not significantly associated with general cognition for children (ES = − 0.33; 95% CI, − 1.02, 0.37), with a median heterogeneity (*I*^2^ = 48.1%) (Fig. 3a). The potential confounding and incomplete outcome data of those two studies are all considered as “probably high” ROB for lacking of important covariates.

#### Language

Nine cohort studies of 2 citations (Guxens et al. 2014; Lin et al. 2014) had assessed the effects of maternal NO_2_ exposure on children’s language. The summary effect estimate was − 0.13 (95% CI, − 0.34, 0.60), and evidence of statistical heterogeneity was low (*I*^2^ = 14.16%) (Fig. 3b). Except for consistent probably high ROB in potential confounding and incomplete data, cohort study in Taiwan also had probably high risk in the assessment of NO_2_ exposure and outcome variable.

#### Psychomotor outcomes

**Fig. 4:**
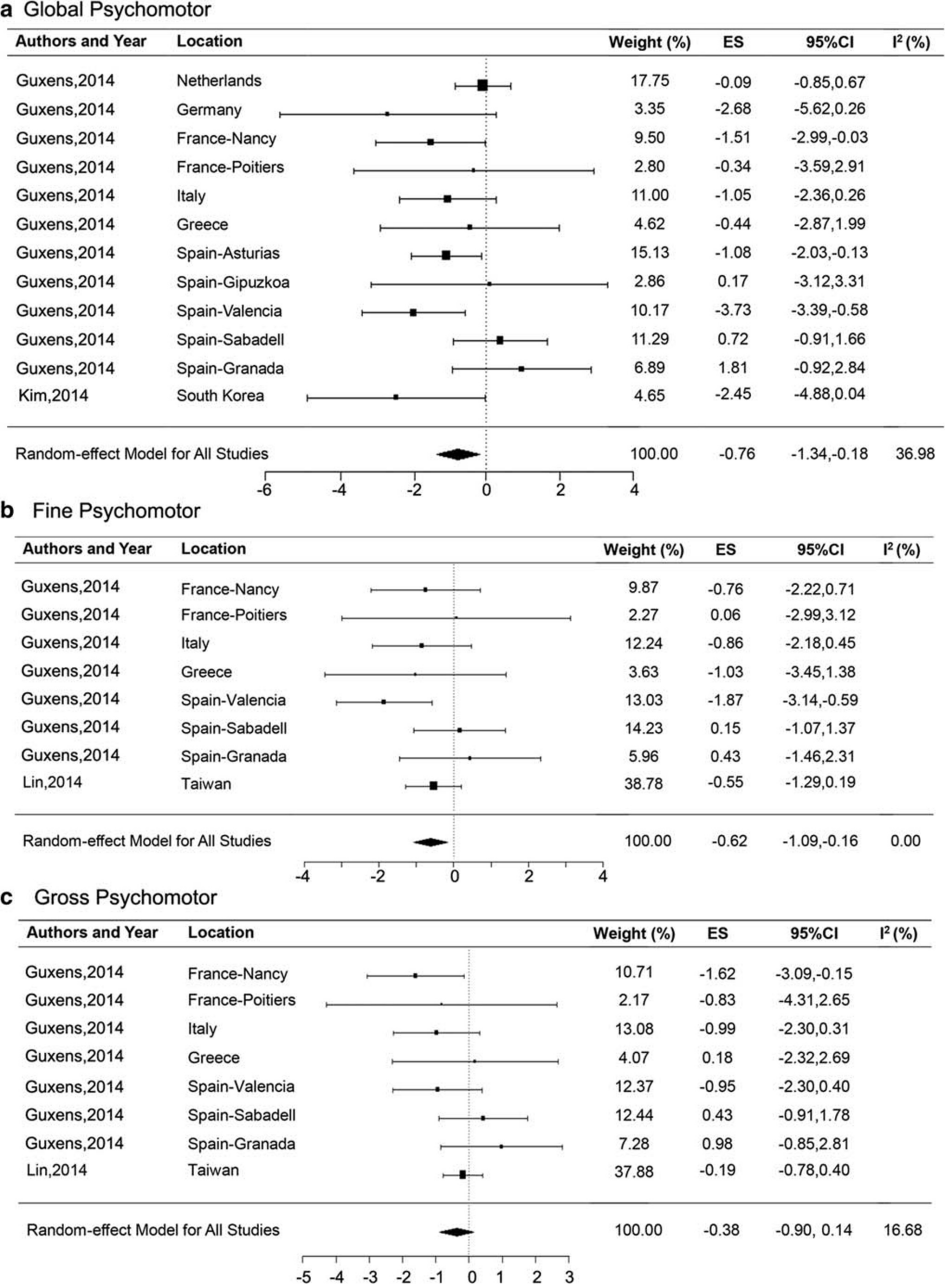
Meta-analysis of maternal exposure to NO_2_ on children’s psychomotor development. ES, effect size; CI, confidence interval; *I*^2^ = percentage of the total variability due to between-areas heterogeneity

Three of the 10 citations included assessed the effects of maternal exposure to NO_2_ on the children’s psychomotor-related outcomes (Guxens et al. 2014; Kim et al. 2014; Lin et al. 2014), including global psychomotor, gross psychomotor, and fine psychomotor. Table 1 summarizes the cognition assessment tools used in each study and specific psychomotor outcome variables. The age of child cognitive testing ranged from less than 6 months to 7 years old. Among them, two citations (Guxens et al. 2014; Kim et al. 2014) (including 12 cohort studies from different regions) estimated the effect of maternal exposure to NO_2_ on children’s global psychomotor. The results of the meta-analysis showed that a 10 μg/m^3^ increase in maternal NO_2_ exposure was significantly associated with children’s global psychomotor with combined ES which was − 0.76, (95% CI, − 1.34, − 0.18), with moderate heterogeneity (*I*^2^ = 36.98%) (Fig. 4a). Moreover, children’s fine psychomotor was also associated significantly with 10 μg/m^3^ increases in maternal NO_2_ exposure (ES = 0.62; 95% CI, − 1.09, − 0.16; *I*^2^ = 0.00%) according to summary estimate of 8 cohort studies among 2 citations (Fig. 4b). But no significant association was found between maternal exposure to NO_2_ and children’s gross psychomotor (ES = − 0.38; 95% CI, − 0.90, 0.14; *I*^2^ = 16.68%) in meta-analysis (Fig. 4c). These studies have a relatively consistent potential high ROB among incomplete data and potential confounding, but low ROB in the assessment of NO_2_ exposure and outcomes. Another study conducted in four regions of Spanish also reported that prenatal exposure to NO_2_ was related with decrease fine motor in boys.

### Synthesized qualitatively in narrative

We developed Table 1 to visually represent the preliminary comprehensive results, which contribute to simply list studies on the effect of NO_2_ on children’s attention, IQ, behavior, and emotion.

#### Attention

Only two studies (Sentis et al. 2017; Yorifuji et al. 2017) estimated the effect of maternal exposure to NO_2_ on children’s attentional problem. A longitudinal survey of 33,890 samples conducted in Japan found that per 10.8 ppm increase in NO_2_ exposure during pregnancy may cause increased risk for “failure to pay attention when crossing a street” for children aged 8 (adjust OR = 1.10; 95% CI, 1.02–1.19). But no obviously impact on the risk for interrupting people and inability to wait his/her turn during play was reported (Yorifuji et al. 2017). But it is worth noting that this study had really high ROB in the assessment of NO_2_ exposure since they assessed maternal NO_2_ exposure level just based on municipality-representative monthly average concentrations. Another cohort study (Sentis et al. 2017) of 1298 children in Spanish showed a lower risk of overall bias. It used the Kiddie-Conners Continuous Performance Test (K-CPT) to assess attention function and found that prenatal exposure to NO_2_ was associated with an impaired standard error of the hit reaction time (HRT(SE)) (increase of 1.12 ms [95% CI, 0.22–2.02] per 10 μg/m^3^ increase in prenatal NO_2_) and increased omission errors (increase of 1.06 ms [95% CI, 1.01–1.11] per 10 μg/m^3^ increase in prenatal NO_2_), which were both indicative inattentiveness.

#### IQ

For IQ, only a cohort study of 719 has been reported (Tzivian et al. 2016). It found that a 10 μg/m^3^ higher NO_2_ exposure during pregnancy was associated with 1.4 fewer points (95% CI, − 2.6, − 0.20) of verbal IQ and 1.4 fewer points (95% CI, − 2.7, − 0.20) of verbal comprehension IQ, but no significant associations with full-scale IQ and performance IQ. However, this study was not control for potential confounders for maternal IQ level.

#### Behavior

Two studies (Ren et al. 2019; Yorifuji et al. 2017) estimated the effect of maternal exposure to NO_2_ on children’s behavior, but the outcome variables between them were different. The study conducted in Japan mainly estimated effects on aggressive behaviors including lying, destroying toys and/or books, hurting other people, and causing disturbances in public. But no significant association was found in this study. Another study of 657 samples using behavior difficult assessed by the Strengths and Difficulties Questionnaire as outcome variable and observed positive associations between exposure to NO_2_ (aOR = 1.204; 95% CI, 1.042, 1.392) and total difficulties, especially in the first trimester (aOR = 1.039; 95% CI, 1.013, 1.066). Both those studies had a probably high ROB. The longitudinal survey in Japan collected main information by mail-related questionnaire, so it caused a probably high ROB in source population representation, the assessment of variables, and incomplete outcome data. Another retrospective survey mainly aimed at kindergarten children and caused high ROB in NO_2_ exposure assessment.

#### Emotion

We only retrieved one study that included emotion as the outcome variable, and it found no significant correlation between maternal exposure to NO_2_ and children’s emotion symptoms (aOR = 0.937; 95% CI, 0.854, 1.108) (Ren et al. 2019). But this study had high ROB in the assessment of NO_2_ exposure since it utilized the air pollution concentrations in kindergarten as the surrogate of maternal exposure during pregnancy.

We were unable to compare our results to those of other systematic reviews because no other reviews have dealt with this topic, as far as we are aware.

## Discussion

Our meta-analysis has found evidence suggestive of a relationship between prenatal exposure to NO_2_ and the development of psychomotor, especially in global psychomotor and fine psychomotor for children. But there is no significant association in language and cognitive development. Through the literature review, only several studies with high ROB have reported possible negative effects, so the relationship between prenatal exposure to NO_2_ and children’s attention function, behavior difference, IQ, and emotion is still unclear. Overall, most studies were rated as “low” or “probably low” risk of bias in most domains, but half of the studies were at probably high ROB in incomplete outcome data and potential confounding.

Our review has found a negative association between prenatal NO_2_ exposure and psychomotor in children, especially in fine psychomotor. This finding was consistent with previous six European birth cohorts (Guxens et al. 2014). Lertxundi et al. also found adverse effects of prenatal NO_2_ exposure on motor scores in women living close to (< 300 m) to metal processing activities (Lertxundi et al. 2015). In addition, a birth cohort conducted in South Korea had suggested that NO_2_ exposure on psychomotor development index (PDI) may be especially stronger at an earlier age since it only found significant adverse effects at 6 months of age, but not at 12 and 24 months (Kim et al. 2014). However, our review found no significant difference while stratified by age (results were not show). Our meta-analysis showed that there was no significant difference between NO_2_ exposure during pregnancy and cognitive development, which was consisted with Guxens’ studies (Guxens et al. 2012; Guxens et al. 2014). However, Lertxundi et al. pointed out different results that prenatal exposure to NO_2_ may decrease children’s mental score, and this negative effect could be higher in the proximity of metal processing plants (Lertxundi et al. 2015). The same negative associations were found in another study after adjusting maternal IQ (Kim et al. 2014). It indicated that maternal IQ might be an important confounding factor between air pollution and neural development in offspring, but was not considered in most included studies. However, there is not enough evidence to support the effect of prenatal NO_2_ exposure on children’s IQ, behavior, and emotion for the lack or contradiction of study. So further studies about utero air pollution exposure on those neural function are generally needed, since some studies suggested that exposure to air pollution during non-pregnancy may cause impairment for IQ and behavior (Calderon-Garciduenas et al. 2015; Sunyer et al. 2015; Tzivian et al. 2016). One study also pointed that NO_2_ exposure during the first trimester was more significantly associated with behavioral (Ren et al. 2019), so the key exposure period should also be addressed in future studies.

Generally speaking, our review found that prenatal exposure to NO_2_ has negative impact in neural development for children and this finding was biologically explicable. It was speculated that impaired immune function, such as oxidative stress (Calderon-Garciduenas 2016; Li et al. 2015, 2016, 2019) and inflammatory responses (Ehsanifar et al. 2019; Ransohoff et al. 2015; Rychlik et al. 2019), may be a potential pathway by which prenatal exposure to NO_2_ may cause an impact on neural functional development. But there was no sufficient evidence indicated the mediated proportion of impaired immune function. In addition, from the perspective of genetics, we speculated that DNA adducts were also a potential pathway, because some studies suggested that DNA adducts in cord blood were associated with children’s behavior and motor scores (Perera et al. 2011; Tang et al. 2008), while it may be higher when maternal exposure to PM_2.5_ and NO_2_ during pregnancy (Pedersen et al. 2009; Pedersen et al. 2015).

Moreover, by meta-analysis, only psychomotor function was found to be impaired. We attributed it to the abnormal release of dopamine in the cerebral cortex. Some animal studies supported evidences, which suggested that a facilitated release of dopamine in the prefrontal cortex or in the striatum was triggered by the diesel exhaust particle exposure during pregnancy (Suzuki et al. 2010; Yokota et al. 2009). The dopamine and noradrenaline systems in the prefrontal cortex had an important role in the control of motor activity through VTA-accumbency-dopamine activity (Oades et al. 1986). In humans, a double-blind randomized crossover study was carried out, in which volunteers were exposed to dilute diesel exhaust or filtered air for 1 h. There was increased activity of the frontal cortex during and after diesel exhaust exposure (Cruts et al. 2008). The frontal cortex controls the actions of the body through its motor areas such as the primary motor or the premotor cortex. Some related animal studies also supported the results of this review. Some studies have found that diesel exhaust particles, black carbon, or NO_2_ exposure during pregnancy decreased the motor function in the offspring mice, attributed this impact to the change of the neurochemical monoamine metabolism of several regions of the brain (Suzuki et al. 2010; Yokota et al. 2009).

To our knowledge, our study is the first meta-analysis and systematic review to estimate comprehensively the effect of prenatal exposure to NO_2_ on children’s neural functional development. Extensive outcome variables were evaluated by our review, including cognition, psychomotor, language, IQ, behavior, and emotion. To avoid repetition bias, we also excluded some estimate values for the same outcome variables that were concomitant in six European cohorts and another article.

One of the limitations of this review is that we cannot carry out meta-analysis on some neural function including attention, behavior, and IQ, since there are few studies with IQ as outcome variables, and the measurement with behavior and attention was inconsistent. However, we summarized and described the previous research results by systematic review and pointed out possible effects and the direction of further research. In addition, as the scope of NO_2_ exposure was not reported in some studies, our review could not conduct stratified analysis based on the regional pollution level although we surmised that the effects in high-polluted areas might be stronger. Publication bias must be considered in our meta-analysis. Scientific investigations that do not find any significant results often fail to be published. The results of egg’s test showed low report bias in each sub-study included in the meta-analysis, but overestimation of the effects value still cannot be ignored.

In our review, we encountered a great variety of methods used for measuring specific neurocognitive abilities. Thus a more homogenous deployment of measurement methods for outcome variable is suggested to future studies. Second, the effect of prenatal air pollution exposure on children’s attention, behavior difference, IQ, and emotion is still unclear and still needs further assessment. In addition, it is necessary to report the average concentration for main pollutants, which can contribute to the subgroup analysis to estimate the effects of prenatal exposure to air pollution on children neural function in high polluted areas. This review only focused on prenatal exposure to NO_2_ and found strong evidence. But air pollution is a mixture contained of various components, including gases (such as NO_2_), particulate matter (PM), metals, and organic compounds that have been found may affect children’s neurodevelopment (Flores-Pajot et al. 2016; Donzelli and Carducci 2019; Donzelli et al. 2019), so it is necessary to conduct more systematic reviews about other pollutants which also may cause adverse impact on neural development.

## Conclusions

The results from our study suggest that prenatal exposure to NO_2_ might be associated with psychomotor, especially in fine psychomotor for children, but not in language and cognitive development. In addition, the relationship between prenatal exposure to NO_2_ and children’s attention, behavior difference, IQ, and emotion is still unclear and requires more confirmation from further research. Based on this, we suggested more homogenous deployment of measurement methods for outcome variable to future studies.

## Electronic supplementary material

ESM 1 (DOCX 19 kb)

ESM 2 (DOCX 42 kb)

ESM 3 (DOCX 17 kb)
