# Supplementary material for: Effects of prenatal exposure to NO2 on children’s neurodevelopment: a systematic review and meta-analysis
Source: Environ Sci Pollut Res Int. 2020 Apr 30;27(20):24786–98. doi: 10.1007/s11356-020-08832-y (PMC7329770; doi:10.1007/s11356-020-08832-y)
Supplement: Supplementary file 1 — (DOCX 19 kb) [file 11356_2020_8832_MOESM1_ESM.docx]

**Appendix A Details of search strategy**

Related articles written in English and published between January 1, 1990 and May 12, 2019 were researched.

The following electronic databases was searched:

- PubMed
- Web of Science
- Embase
- Cochrane Central Register of Controlled Trials

Specific search strategies of each database are as follows:

**PubMed strategy: 1857 citations**

(“maternal exposure”[MeSH Terms] OR maternal [All Fields]) OR (pregnancy [MeSH Terms] OR pregnancy [All Fields]) OR (“pregnant women”[MeSH Terms] OR pregnant women [All Fields]) OR prenatal [All Fields] OR gestation [All Fields]

AND

("nitrogen dioxide"[MeSH Terms] OR nitrogen dioxide[All Fields]) OR (nitrogen oxides"[MeSH Terms] OR nitrogen oxides[All Fields]) OR ("traffic-related pollution"[MeSH Terms] OR traffic-related pollution[All Fields]) OR ("air pollution"[MeSH Terms] OR air pollution[All Fields]) OR ("air pollutants"[MeSH Terms] OR air pollutants[All Fields]) OR nitrogen peroxide[All Fields] OR NO_2_[All Fields]OR Vehicle Pollution[All Fields]

AND

(cognition[MeSH Terms] OR cogniti*[All Fields]) OR (language[MeSH Terms] OR language[All Fields]) OR (behavior[MeSH Terms] OR behavior*[All Fields]) OR intelligence[MeSH Terms] OR intelligence[All Fields]) OR (attention[MeSH Terms] OR attention[All Fields]) OR (emotion[MeSH Terms] OR emotion[All Fields]) OR IQ [All Fields] OR psychomotor[All Fields] OR motor[All Fields] OR neural [All Fields] OR neurodevelopment[All Fields]

AND

(child [MeSH Terms] OR child*[All Fields]) OR (infant [MeSH Terms] OR infant*[All Fields]) OR offspring [All Fields]

**Embase: 685 citations**

('maternal'/exp OR 'pregnancy'/exp OR 'pregnant woman'/exp OR 'prenatal'/exp) OR maternal OR pregnancy OR (pregnant AND woman) OR prenatal OR gestation

AND

('nitrogen dioxide'/exp OR 'nitrogen oxide'/exp OR 'traffic pollution'/exp OR 'air pollution'/exp OR 'air pollutant'/exp) OR ((nitrogen AND dioxide) OR (nitrogen AND oxide*) OR (air AND pollution) OR (air AND pollutant*) OR NO_2_ OR (traffic-related AND pollution) OR (nitrogen AND peroxide) OR (vehicle AND pollution))

AND

('cognition'/exp OR 'language'/exp OR 'behavior'/exp OR 'intelligence'/exp OR 'attention'/exp OR 'emotion'/exp OR 'psychomotor development'/exp) OR (cognit* OR 'language* OR Behavior* OR intelligence OR attention OR emotion OR IQ OR psychomotor OR motor OR neural OR neurodevelopment)

AND

('child'/exp OR 'infant'/exp) OR (child OR children* OR infant* OR offspring*)

AND

[1-1-1990]/sd NOT [12-5-2019]/sd

**Web of Science: 1218 citations**

TS=(pregnancy OR "pregnant women" OR "pregnant woman" OR maternal OR prenatal OR gestation)

AND

TS=("nitrogen dioxide" OR "nitrogen oxides" OR (air AND pollut*) OR (traffic-related AND pollut*) OR (vehicle AND pollut*) OR "nitrogen peroxide" OR NO_2_)

AND

TS=(cogniti* OR language* OR behavior* OR intelligence OR attention OR emotion* OR IQ OR psychomotor OR motor OR neural OR neurodevelopment)

AND

TS=(child OR children* OR infant* OR offspring*)

**Cochrane Central Register of Controlled Trials: 61 citations**

pregnancy OR "pregnant women" OR "pregnant woman" OR maternal OR prenatal OR gestation[All Text]

AND

"nitrogen dioxide" OR "nitrogen oxides" OR (air AND pollut*) OR (traffic-related AND pollut*) OR (vehicle AND pollut*) OR "nitrogen peroxide" OR NO_2_[All Text]

AND

cogniti* OR language* OR behavior* OR intelligence OR attention OR emotion* OR IQ OR psychomotor OR motor OR neural OR neurodevelopment[All Text]

AND

child OR children* OR infant* OR offspring*[All Text]
