# Supplementary material for: Effects of prenatal exposure to NO2 on children’s neurodevelopment: a systematic review and meta-analysis
Source: Environ Sci Pollut Res Int. 2020 Apr 30;27(20):24786–98. doi: 10.1007/s11356-020-08832-y (PMC7329770; doi:10.1007/s11356-020-08832-y)
Supplement: Supplementary file 3 — (DOCX 17 kb) [file 11356_2020_8832_MOESM3_ESM.docx]

**Appendix C Formula of the transformation of estimated value**

1. The transformation of 95% confidence interval (CI) and standard error (SE):

$95\%CI=\beta\pm1.96 SE$ Eq. (A.1)

1. The transformation of μg/m^3^ and ppb for the concentration of NO_2_:

$C=C^{'}*\frac{M}{22.4}*\frac{273}{273+T}*\frac{\mathrm{Pa}}{101325}$ Eq. (A.2)

C means concentration of NO_2_ in mg/m^3^, 1 mg/m^3^ equals 1000 μg/m^3^.

C’ means concentration of NO_2_ in ppm, 1 ppm equals 1000ppb.

M means molecular weight of NO_2_, which equals 46.1.

T means temperature, which is assumed to 25 ℃.

Pa means atmospheric pressure, which is assumed to be standard atmospheric pressure (101.325 KPa).

After the calculation, we figured out that 1 μg/m^3^ equals 1.885ppb and 100ppb equals 1.8851μg/m^3^.
